# Social Stimulus Causes Aberrant Activation of the Medial Prefrontal Cortex in a Mouse Model With Autism-Like Behaviors

**DOI:** 10.3389/fnsyn.2018.00035

**Published:** 2018-10-12

**Authors:** Antonella Pirone, Jonathan M. Alexander, Jenny B. Koenig, Denise R. Cook-Snyder, Medha Palnati, Robert J. Wickham, Lillian Eden, Neha Shrestha, Leon Reijmers, Thomas Biederer, Klaus A. Miczek, Chris G. Dulla, Michele H. Jacob

**Affiliations:** Department of Neuroscience, Sackler School of Biomedical Sciences, Tufts University School of Medicine, Boston, MA, United States

**Keywords:** autism, social behavior, β-catenin, APC, parvalbumin, medial prefrontal cortex, infralimbic

## Abstract

Autism spectrum disorder (ASD) is a highly prevalent and genetically heterogeneous brain disorder. Developing effective therapeutic interventions requires knowledge of the brain regions that malfunction and how they malfunction during ASD-relevant behaviors. Our study provides insights into brain regions activated by a novel social stimulus and how the activation pattern differs between mice that display autism-like disabilities and control littermates. Adenomatous polyposis coli (APC) conditional knockout (cKO) mice display reduced social interest, increased repetitive behaviors and dysfunction of the β-catenin pathway, a convergent target of numerous ASD-linked human genes. Here, we exposed the mice to a novel social vs. non-social stimulus and measured neuronal activation by immunostaining for the protein c-Fos. We analyzed three brain regions known to play a role in social behavior. Compared with control littermates, APC cKOs display excessive activation, as evidenced by an increased number of excitatory pyramidal neurons stained for c-Fos in the medial prefrontal cortex (mPFC), selectively in the infralimbic sub-region. In contrast, two other social brain regions, the medial amygdala and piriform cortex show normal levels of neuron activation. Additionally, APC cKOs exhibit increased frequency of miniature excitatory postsynaptic currents (mEPSCs) in layer 5 pyramidal neurons of the infralimbic sub-region. Further, immunostaining is reduced for the inhibitory interneuron markers parvalbumin (PV) and somatostatin (SST) in the APC cKO mPFC. Our findings suggest aberrant excitatory-inhibitory balance and activation patterns. As β-catenin is a core pathway in ASD, we identify the infralimbic sub-region of the mPFC as a critical brain region for autism-relevant social behavior.

## Introduction

Autism spectrum disorder (ASD) is diagnosed in 1 out of every 59 children and the prevalence continues to rise. Large-scale genetic screens of families with ASD have identified hundreds of risk genes, and their known roles suggest convergence on aberrant synaptic function as a core cause of ASD (Gilman et al., 2011; Zoghbi and Bear, 2012). Synaptic dysfunction as a cause of the hallmark behavioral changes in ASD (reduced social interest, increased repetitive behaviors and reduced communication) is thought to stem from an imbalance between excitatory and inhibitory activity in synaptic transmission and brain circuits (Lee et al., 2017). As strong support, several ASD-linked gene mutations result in E/I imbalance, including mutations in the inhibitory and excitatory neurotransmitter receptors (GABARs and NMDARs) and neuroligin and neurexin synaptic adhesion molecules (Jamain et al., 2003; Barnby et al., 2005; Feng et al., 2006; Szatmari et al., 2007; Glessner et al., 2009; O’Roak et al., 2011). However, the brain regions and cell types that mediate social and repetitive behaviors remain incompletely defined in the healthy brain, as do the brain regions which malfunction in autism-relevant behaviors.

Social behavior has been linked to at least three brain regions, the medial prefrontal cortex (mPFC), piriform cortex and medial amygdala (Baron-Cohen et al., 2000; Ferguson et al., 2001; Richter et al., 2005; Kim et al., 2015; Li et al., 2017). Optogenetically induced E/I imbalance, to increase pyramidal neuron excitation in the mPFC, leads to reduced social interest relative to non-stimulated mice (Yizhar et al., 2011). Social deficits in the *CNTNAP2* mouse model are acutely rescued by either optogenetically increasing excitation of GABAergic parvalbumin (PV) expressing interneurons or decreasing excitation of glutamatergic pyramidal neurons in the mPFC (Selimbeyoglu et al., 2017). Further, several individuals with ASD and multiple mouse models of ASD display reductions in PV expression levels or interneuron number in this brain region (Rubenstein and Merzenich, 2003; Gogolla et al., 2009; Peñagarikano et al., 2011; Saunders et al., 2013; Jeevakumar et al., 2015; Wöhr et al., 2015; Filice et al., 2016; Hashemi et al., 2017). Our study tests for aberrant activation patterns in social brain regions in another mouse model with autism-like behaviors, to gain critical insights into convergent brain regions as potential therapeutic targets with relevance to several ASD-linked genes.

Human genetic studies show a strong association between ASD and β-catenin network malfunction, including mutations in *CTNNB1* (β-catenin) itself, *Shank3*, a β-catenin interacting protein, and *CHD8*, a regulator of β-catenin’s transcriptional activity and the leading cause of non-syndromic ASD (Iossifov et al., 2012; Neale et al., 2012; O’Roak et al., 2012; Talkowski et al., 2012; Krumm et al., 2014; Durak et al., 2016; Cho et al., 2017). β-catenin has dual roles in neurons: interacting with synaptic cadherins to dynamically regulate synaptic function and mediating transcription of canonical Wnt target genes. Both pathways are essential for normal synaptic density, plasticity and function. Adenomatous polyposis coli (APC) protein is the major negative regulator of β-catenin levels as an essential component of the multi-molecular degradation complex (Stamos and Weis, 2013), and mutations in *APC* have also been linked to ASD (Barber et al., 1994; Zhou et al., 2007). Further, APC is enriched at excitatory postsynaptic sites, links synaptic components to the cytoskeleton, and regulates actin and microtubule cytoskeleton dynamics (Matsumine et al., 1996; Yanai et al., 2000; Temburni et al., 2004; Zhou et al., 2004; Rosenberg et al., 2008, 2010; Yokota et al., 2009; Chen et al., 2011; Mohn et al., 2014). Additionally, APC is an RNA-binding protein, and its interactome includes several mRNAs that link to brain development and ASD (Preitner et al., 2014).

To elucidate the brain regions that malfunction during autism-like behaviors caused by dysregulation of β-catenin and APC, we used APC conditional knockout (cKO) mice. Our conditional genetic manipulation (CamKIIα-Cre mediated deletion of APC) predominantly targets excitatory glutamatergic neurons. APC loss causes excessive increases in β-catenin and Wnt target gene expression which are known to regulate synaptic activity, spine density and plasticity (Mohn et al., 2014). Compared with control littermates, APC cKO mice display reduced social interest and increased repetitive behaviors, as well as cognitive impairments and spontaneous seizures, which are often co-morbid with autism (Mohn et al., 2014; Pirone et al., 2017). APC cKOs also exhibit increases in spine density and miniature excitatory postsynaptic currents (mEPSCs) frequency in pyramidal neurons of hippocampal CA1 and layer 5 sensorimotor cortex, suggesting enhanced excitation.

In the present study, we use exposure to a novel social vs. non-social stimulus and look for differences in the neuronal activation pattern in three different social brain regions of APC cKOs relative to control littermates. We show aberrant responses within distinct sub-regions of the mPFC of APC cKOs. We also show changes in synaptic activity (mEPSC frequency) and expression levels of both PV and somatostatin (SST), key inhibitory interneuron subtypes known to regulate activity. Our findings contribute to the emerging evidence for a convergent role of the mPFC in autism-relevant social behaviors. Further, we provide novel insights into the infralimbic cortex as the critical brain region altered by malfunction of the β-catenin pathway that links to numerous human ASD genes.

## Materials and Methods

### Animals

APC cKO mice were generated as previously described (Mohn et al., 2014). All experiments included control littermates that were floxed APC, Cre-recombinase negative and/or control APC, Cre-recombinase positive. The mice were evaluated at adult ages (~3 months old), and equal numbers of males and females were used for all experiments. Additionally, for a subset of the c-Fos immunostaining experiments, APC cKO mice were crossed with GAD67-EGFP (line G42) mice (CB6-Tg (Gad1-EGFP) G42Zjh/J, Jackson Laboratory) for fluorescent labeling of the PV-expressing subset of GABAergic neurons (Chattopadhyaya et al., 2004). This study was carried out in accordance with the recommendations of the NIH guidelines and the Tufts University Institutional Animal Care and Use Committee. The protocol was approved by the Tufts University Institutional Animal Care and Use Committee.

### Social vs. Non-social Behavioral Stimulation Paradigm

Animals were single-housed on a reverse-light dark cycle 1 week prior to the beginning of habituation. During the habituation period, the mice were exposed to the investigators gloved hand briefly entering the cage, the hand was removed for 3 min, and the investigators hand was briefly inserted into the cage once again. This was done once daily for each mouse for 5 days in order to habituate the mice to the presence of the investigator’s hand in the cage when inserting either the novel object or novel mouse in the probe trial. On probe day (day 6), the mice were either subjected to a 3-min exposure to a novel object or juvenile (~21 day old) male mouse introduced into the cage. Ninety minutes later, the mice were transcardially perfused with 4% paraformaldehyde in phospho-buffered saline (PBS) and processed for c-Fos immunostaining as a marker of neuronal activation.

### Immunostaining

Following perfusion, brains were post-fixed in 4% paraformaldehyde in PBS overnight at 4°C. Brains were then transferred to 30% sucrose in PBS plus 0.1% sodium azide for 24–72 h, before being preserved in isopentane cryoprotectant, frozen and sectioned by cryostat at 20 μm thickness. For the mice exposed to the novel social or non-social stimulus, coronal sections were incubated in 5% normal goat serum in PBST for 1 h to block non-specific binding and immunostained for c-Fos (Santa Cruz, sc-52 mAB, 1:10,000 dilution) and neuronal nuclei (NeuN; Millipore, #MAB377, 1:2,000). Additional APC cKO and control littermate mice were processed similarly and immunostained for PV (Swant, PV27, 1:2,000) and SST (Millipore, MAB354, 1:1,000) staining. APC cKO-GAD67-EGFP mice and their control littermates were also processed similarly and immunostained for c-Fos, to determine its cellular distribution relative to the EGFP marked PV interneurons. Sections used for c-Fos and NeuN were imaged with a Zeiss Axioskop epifluorescence microscope (Carl Zeiss Microscopy, Jena, Germany). Sections used for PV and SST immunostaining and for c-Fos, relative to GAD67-EGFP, were imaged with Keyence 3D epifluorescence microscope (BZ-X series). Regions of interest (ROIs) demarcating the infralimbic and prelimbic sub-regions of the mPFC were assigned to the sections at Bregma positions which were matched between the genotypes for comparisons. Quantification of c-Fos+, NeuN+, PV+ and SST+ cells was performed in ImageJ.

### Electrophysiological Recordings

Acute brain slices were prepared from adult APC cKO mice and control littermates as described previously (Pirone et al., 2017). Briefly, mice were anesthetized with isoflurane and euthanized. The brain was rapidly dissected and immediately placed into ice-cold cutting solution (2.5 mM KCl, 1.25 mM NaH2PO_4_, 10 mM MgSO_4_, 0.5 mM CaCl_2_, 11 mM glucose, 234 mM sucrose, 26 mM NaHCO_3_) equilibrated with 95% O_2_:5% CO_2_. The brain was glued to the stage of a Leica Vibratome VT 1200s, 300-μm thick coronal slices were cut, hemisected and placed into artificial cerebrospinal fluid (aCSF: 126 mM NaCl, 2.5 mM KCl, 1 mM MgSO_4_, 2 mM CaCl_2_, 10 mM glucose, 26 mM NaHCO_3_) in a recovery chamber equilibrated with 95% O_2_:5% CO_2_ at 32°C. After 1 h, the slices were kept in the recovery chamber at room temperature and used for electrophysiological recording within 6 h of slicing.

Whole-cell recording was used to measure the frequency and amplitude of mEPSCs in layer 5 pyramidal neurons of the infralimbic mPFC. The slices were placed in the recording chamber of an Olympus BX51 microscope, held in place with gold wires, and continuously perfused (2 mL/min) with aCSF containing 1 μM tetrodotoxin (TTX; to block sodium channels) and equilibrated with 95% O_2_:5% CO_2_ at 32°C. The borosilicate glass electrodes (2–5 MΩ resistance) were filled with internal solution (140 mM CsMS, 10 mM HEPES, 5 mM NaCl, 200 μM EGTA, 5 mM QX314-Br, 1.8 mM MgATP, 300 μM NaGTP, pH 7.25). Cells were voltage-clamped at −70 mV. Data were collected with an Axon Multiclamp 700 B amplifier, Digidata 1440A digitizer, and pClamp software. Cells were accepted for analysis if the access resistance varied <20% during the 2-min recording time. mEPSC recordings were analyzed using Clampfit (Axon Instruments) and MiniAnalysis (Synaptosoft). The investigator was blinded to genotype during analysis. The values for event frequency, amplitude, rise time, decay time and half width were averaged between cells in each group, and compared with a 2-sample Student’s *t*-test. Cumulative distributions of inter-event interval and event amplitude were generated using a random event sampling approach to ensure that data from more active cells were not overrepresented relative to cells with fewer events. To accomplish this, a custom-written MATLAB script was utilized to randomly select 50 events from each recorded cell. That number of events was selected to ensure all cells would have an equal number of events included in the group population. Within a genotype, randomly selected events from each cell were pooled to generate a single distribution for that genotype. Distributions of events were then compared using a 2-sample Kolmogorov-Smirnov (K-S) test. For K-S tests, α = 0.001 to avoid false positives associated with large degrees of freedom.

### Statistical Analysis

All results are listed as the arithmetic mean ± SEM and the statistical tests used for each experiment are indicated within the text. Sample size was determined looking for a large effect (>0.5) at 80% power. For immunostaining statistics, the average count/area across slices for each animal was used as a single data point, and the resulting power and effect size are listed for significant results.

## Results

### APC cKO Mice Demonstrate Increased Activation of Pyramidal Neurons of the Infralimbic Prefrontal Cortex in Response to a Novel Social Stimulus

**Figure 1:**
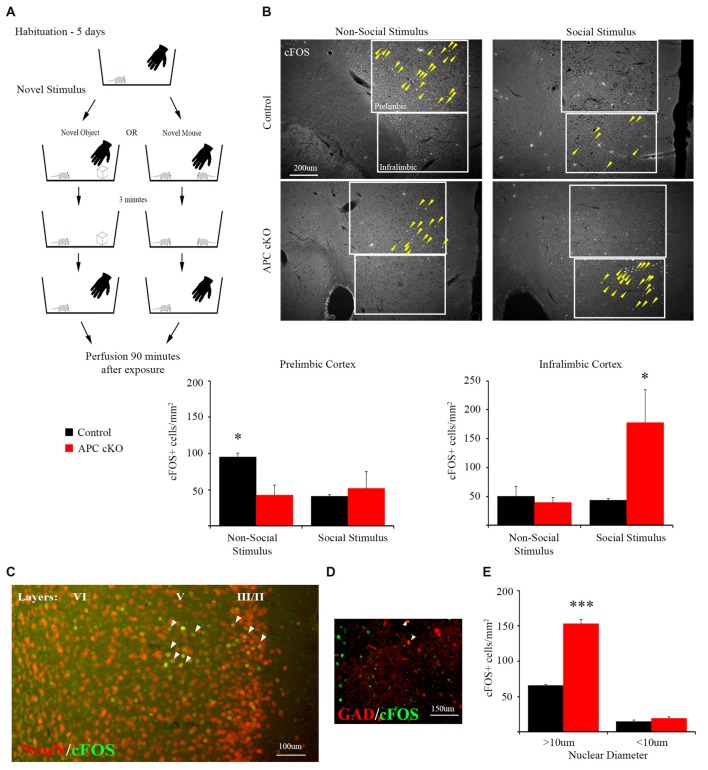
A novel social stimulus causes aberrant c-Fos activation in the medial prefrontal cortex (mPFC) in the adenomatous polyposis coli (APC) conditional knockout (cKO) mouse. **(A)** Schematic for social vs. non-social exposure experiment. During the 5-day habituation period, the mouse is singly housed and exposed to the investigator’s hand twice, separated by 3 min, for 1 s each time. During the exposure trial, the mice are separated into one of two test groups: exposure to either a novel, non-social object for 3 min before it is removed; or exposure to a novel, juvenile mouse for 3 min before it is removed. Ninety minutes after removal of the novel stimulus, the mice were perfused and brain sections containing the mPFC were immunostained for c-Fos as a marker of neuronal activity. **(B)** Representative images and quantification of c-Fos positive cells in prelimbic and infralimbic sub-regions (delineated by box lines) of the mPFC shows that social exposure induces increased activation of neurons in the infralimbic cortex of APC cKO mice. In contrast novel object exposure causes less activation in the prelimbic sub-region in APC cKOs, compared with control littermates (arrowheads on c-Fos positive cells are included to highlight the significantly different activation patterns, for the novel object: *n* = 3 mice for each genotype, for the novel mouse: *n* = 3 mice for each genotype, two slices per mouse; *p < 0.05). **(C)** Representative image of double labeling showing that the c-Fos immuno-positive cells (green) also stain for neuronal nuclei (NeuN; red) [overlap (yellow) indicated by arrowheads]. **(D)** The c-Fos staining is detected in relatively few GAD67-EGFP marked parvalbumin (PV) interneurons (*n* = 3 control mice, six slices; APC cKO-GAD67-EGFP six mice, 12 slices [overlap (yellow) indicated by arrowheads]). Note that, to make the color for c-Fos consistent across the images shown in **(C)**, we switched the GAD67-EGFP to red and the c-Fos to green in this panel. **(E)** Quantification of the c-Fos positive neurons (*n* = 3 mice for each genotype, ***p < 0.001), identified as pyramidal cells given their large nuclear diameter (>10 μm), NeuN staining and localization in layers 2/3 and 5 of the infralimbic sub-region, or as interneurons (<10 μm nuclear diameter) and NeuN or GAD67-EGFP positive.

APC cKO mice display autism-like behaviors, consisting of reduced social interest and increased repetitive behaviors, relative to control littermates (Mohn et al., 2014). To gain insights into the particular brain regions that may mediate the abnormal social behaviors, we tested for aberrant activation patterns in APC cKOs, compared with control littermates, using a social/non-social stimulus paradigm (Figure 1A). We used immunostaining for the c-Fos protein, as a marker of neuronal activation, and co-stained with NeuN protein. We examined three brain regions implicated in social behavior, the piriform cortex, medial amygdala and mPFC.

Compared with control littermates, the APC cKO mice exhibited normal levels of activation in both the piriform cortex and medial amygdala in response to a novel social stimulus, based on the density of c-Fos immuno-positive neurons (*piriform cortex*: c-Fos positive cells/mm^2^, *n* = 3 control mice, 13 slices; APC cKO three mice, 10 slices, Two-way ANOVA Interaction *F*_(1,8)_ = 1.201, *p* = 0.3049, Genotype *F*_(1,8)_ = 0.061, *p* = 0.8119, Stimulus *F*_(1,8)_ = 22.8, *p* = 0.0014; non-social stimulus: Control 173.85 ± 17.47, APC cKO 136.73 ± 22.48; social stimulus: Control 275.60 ± 20.15, APC cKO 299.11 ± 42.94, *p* = 0.810 Sidak adjusted *t*-test; *medial amygdala*: c-Fos positive cells/mm^2^, *n* = 3 control mice, 11 slices; APC cKO three mice, 10 slices, Two-way ANOVA Interaction *F*_(1,8)_ = 0.2878, *p* = 0.606, Genotype *F*_(1,8)_ = 0.487, *p* = 0.505, Stimulus *F*_(1,8)_ = 43.31, *p* = 0.002; non-social stimulus: Control 71.64 ± 14.08, APC cKO 66.40 ± 14.00; social stimulus: Control 302.55 ± 29.96, APC cKO 262.50 ± 54.02).

In contrast, APC cKOs showed increased activation in the mPFC, in particular in the infralimbic sub-region after exposure to the novel social stimulus (Figure 1B, C-Fos positive cells/mm^2^, *n* = 3 mice each genotype, two slices per mouse, Two-way ANOVA Interaction *F*_(1,8)_ = 5.767, *p* = 0.0431, Genotype *F*_(1,8)_ = 4.129, *p* = 0.0766, Stimulus *F*_(1,8)_ = 4.645, *p* = 0.0633; Control 42.97 ± 3.74, APC cKO 177.48 ± 57.56, *p* = 0.0276 Sidak adjusted *t*-test, power = 91%, *d* = 1.39). Using a separate cohort of mice, we have validated the significant increase in c-Fos neurons in the infralimbic of APC cKOs, compared with control littermates, in response to the social stimulus (c-Fos positive cells/mm^2^, *n* = 3 control mice, six slices; APC cKO three mice, five slices; Control 47.41 ± 6.77, APC cKO 90.93 ± 20.15, *p* = 0.0427 Student’s *t*-test). Further, we identify the majority of c-Fos positive cells as pyramidal neurons based on their being localized predominantly in layers 2/3 and 5 of the infralimbic cortex, having a nuclear diameter >10 μm, and staining positive for the NeuN neuronal marker (Figures 1C,E, C-Fos positive cells/mm^2^, *n* = 3 mice each genotype, Two-way ANOVA Interaction *F*_(1,4)_ = 205.6, *p* = 0.0001, Genotype *F*_(1,4)_ = 163.7, *p* = 0.0002, cell diameter *F*_(1,4)_ = 1010, *p* < 0.0001; >10 μm: Control 64.03 ± 1.31, APC cKO 150.27 ± 5.58, *p* < 0.001 Sidak adjusted *t*-test). In comparison, interneurons are a much smaller proportion of the activated cells; as all of the GAD67-EGFP positive cells had a nuclear diameter <10 μm, we quantified the c-Fos positive neurons of this size that were either NeuN positive (cohort 1) or GAD67-EGFP positive Figures 1D,E (cohort 2; <10 μm: Control 14.37 ± 1.73, APC cKO 18.95 ± 2.36, *p* = 0.570 Sidak adjusted *t*-test). This finding is consistent with reports that excessive activation of pyramidal neurons in the mPFC leads to reduced social interactions (Yizhar et al., 2011; Jeevakumar et al., 2015; Selimbeyoglu et al., 2017). However, we cannot rule out the possibility that other brain regions involved in social behavior, such as the septum and BNST, implicated in aggression and dominance behaviors, may also exhibit an aberrant response to the novel social stimulus in the APC cKOs.

Additionally, compared to the APC cKOs, control littermates display significantly greater activation in response to the non-social stimulus in the mPFC prelimbic sub-region (Figure 1B; c-Fos positive cells/mm^2^, *n* = 3 mice each genotype, two slices per mouse, Two-way ANOVA Interaction *F*_(1,8)_ = 5.575, *p* = 0.0459, Genotype *F*_(1,8)_ = 2.384, *p* = 0.1612, Stimulus *F*_(1,8)_ = 2.672, *p* = 0.1408; Control 95.27 ± 5.39, APC cKO 42.07 ± 13.94, *p* = 0.0486 Sidak adjusted *t*-test, power = 99.9%, *d* = 1.59). The density of NeuN immunostained cells was not significantly different between APC cKOs with control littermates in either the infralimbic or prelimbic (*n* = 6 mice per genotype, two slices per animal, NeuN+ cells/mm^2^; *prelimbic*: Control 2,408.0 ± 54.9, APC cKO 2,313.7 ± 33.9, *p* = 0.175 Student’s *t*-test; *infralimbic*: Control 2,295.1 ± 52.5, APC cKO 2,344.5 ± 72.0, *p* = 0.591 Student’s *t-test*).

These distinct aberrant activation patterns in the two different sub-regions of the mPFC suggest altered cognitive perception of both social and non-social stimuli in APC cKO mice. Further, we identify distinct sub-regions within the mPFC as responding selectively to social (infralimbic) vs. non-social (prelimbic) stimuli in both the healthy control littermates and in the APC cKOs with autism-relevant behaviors.

### APC cKO Mice Exhibit Increased mEPSC Frequency in the Infralimbic Prefrontal Cortex

Based on the increased c-Fos activation in the infralimbic mPFC after social stimulation of APC cKOs, we assessed excitation in the major projection neurons, the layer 5 pyramidal cells. We measured the frequency and amplitude of mEPSCs by whole cell recordings in acute cortical slices from both APC cKO mice and littermate controls. The recorded neurons were identified as excitatory pyramidal neurons based on their morphology and biophysical properties. Using infrared differential interference contrast (DIC) microscopy, we targeted the neurons with large diameter, pyramidal-shaped somas. In comparison, the inhibitory interneurons have small, round somas, and represent <20% of the neurons in the cortex. Additionally, we quantified the membrane resistance (*R*_m_) of each recorded neuron to further identify the subtype. GABAergic fast-spiking interneurons tend to have low input resistance *R*_m_ values near 100 MΩ (Kawaguchi and Kubota, 1997), while layer 5 pyramidal neurons tend to have *R*_m_ values closer to 300 MΩ. The membrane resistance of the recorded neurons is consistent with known values for excitatory pyramidal neurons (WT = 303.4 ± 31.7 MΩ; cKO = 288.9 ± 29.5 MΩ).

**Figure 2:**
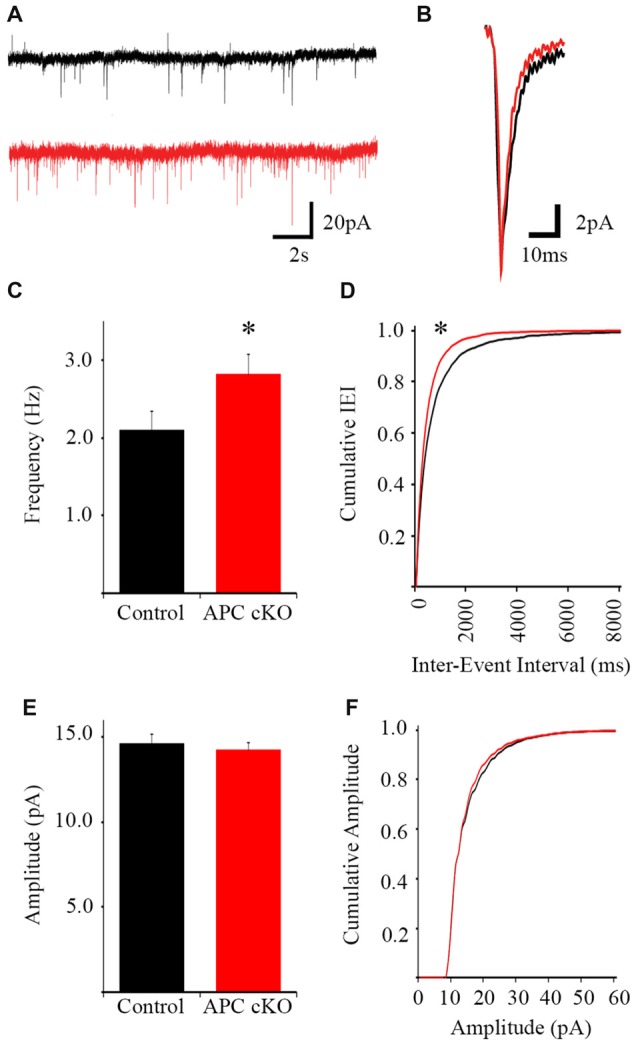
Increased miniature excitatory postsynaptic current (mEPSC) frequency in pyramidal neurons of the infralimbic mPFC in APC cKO mice. Examples of 20-s segments **(A)** and individual events **(B)** of 2-min long traces from control littermate and APC cKO neurons (*n* = 26 control cells, 30 APC cKO cells, from four animals per genotype). **(C)** The frequency of mEPSCs is increased in APC cKOs relative to controls (*p < 0.05). **(D)** Cumulative distribution of the inter-event interval shows a leftward shift in the APC cKO (corresponding with shorter IEI, *p < 0.001). **(E)** There is no significant difference in event amplitude or **(F)** cumulative distribution of event amplitude between the two groups.

The pyramidal neurons were voltage clamped at −70 mV to isolate excitatory currents and TTX was included in the perfusate to eliminate action potential-dependent synaptic events. APC cKOs exhibit increased average mEPSC frequency (Control 2.09 Hz ± 0.24, APC cKO 2.61 Hz ± 0.04, *p* = 0.044 Student’s *t*-test) and cumulative mEPSC frequency distribution (K-S test *p* = 2.04E-8) relative to littermate controls (Figures 2A–D). However, we observed no significant changes in either average mEPSC amplitude (Figure 2E; Control 14.64 pA ± 0.54, APC cKO 14.44 pA ± 0.44, *p* = 0.789 Student’s *t*-test) or cumulative mEPSC amplitude distribution (Figure 2F; K-S test, *p* = 0.779) between genotypes. Rise time (Control 2.76 ms ± 0.07, APC cKO 2.75 ms ± 0.06, *p* = 0.837 Student’s *t*-test), decay time (Control 6.15 ms ± 0.32, APC cKO 6.05 ms ± 0.21, *p* = 0.778 Student’s *t*-test), and half-width (Control 3.92 ms ± 0.22, APC cKO 3.89 ms ± 0.16, *p* = 0.923 Student’s *t*-test) were also similar between APC cKOs and control littermates. Thus, layer 5 pyramidal neurons of the infralimbic receive an increased number of action potential-independent excitatory inputs in APC cKO mice. Consistent with this finding, we have previously found increases in both mEPSC frequency and spine density of pyramidal neurons in layer 5 sensorimotor cortical and CA1 hippocampal regions of APC cKO mice, compared with control littermates (Mohn et al., 2014; Pirone et al., 2017).

### Reduced PV and SST Interneuron Staining in the APC cKO mPFC

**Figure 3:**
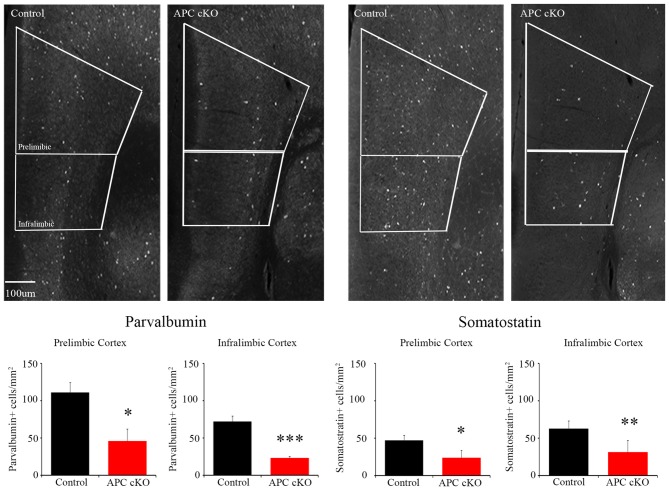
APC cKO mice show reduced numbers of PV and somatostatin (SST) immuno-positive interneurons in the mPFC. Immunostaining in the mPFC shows reduced numbers of PV and SST immunostained cells in both the prelimbic and infralimbic sub-regions (*n* = 3 animals, two slices per mouse for each genotype; *p < 0.05, **p < 0.01, ***p < 0.001).

Based on these activation pattern and excitatory synapse changes, we hypothesized that inhibitory systems may also be altered in the mPFC of the APC cKOs. As a first step to test this, we analyzed the abundance of inhibitory interneurons in the mPFC of APC cKOs. We used immunostaining to examine the expression levels of two inhibitory interneuron subtypes, PV and SST. We observed decreases in the relative density of both PV- and SST- immuno-positive cells in the infralimbic sub-region of APC cKOs, compared to littermate controls (Figure 3; cells/mm^2^ PV: Control 71.83 ± 7.79, APC cKO 22.85 ± 2.61, *p* = 0.00014, Student’s *t*-test, power = 100%, *d* = 1.38; SST: Control 63.01 ± 9.70, APC cKO 31.33 ± 15.58, *p* = 0.0031 Student’s *t*-test, power = 68.5%, *d* = 1.19. We found similar decreases in both PV and SST in the prelimbic sub-region (Figure 3; cells/mm^2^ PV: Control 110.84 ± 13.30, APC cKO 45.90 ± 16.01, *p* = 0.012 Student’s *t*-test, power = 99.3%, *d* = 1.67; SST: Control 47.26 ± 6.44, APC cKO 23.85 ± 9.65, *p* = 0.013 Student’s *t*-test, power = 81.4%, *d* = 1.29). Our results suggest that both excitatory and inhibitory changes cause an excitatory/inhibitory imbalance in the mPFC that likely underlies the reduced social interest of APC cKO mice.

## Discussion

APC cKO mice exhibit malfunction of β-catenin/Wnt signaling, a core pathway that links to numerous human ASD risk genes. Our major finding is that exposure to a novel social stimulus causes excessive activation of neurons in the infralimbic sub-region of the mPFC in these mice. Further, in this sub-region, APC cKOs display increased synaptic activity (frequency of mEPSCs) in layer 5 pyramidal neurons and reduced expression levels of PV and SST, markers of two inhibitory interneuron subtypes. Consistent with these changes, APC cKOs show autism-like behaviors, both reduced social interest and increased repetitive behaviors, as well as cognitive impairments and seizures, co-morbidities often seen with ASD in humans (Mohn et al., 2014; Pirone et al., 2017). Our results contribute to the emerging evidence that the mPFC is a key brain region for manifesting autism-relevant social behavior changes. Importantly, we extend the current knowledge of the mPFC’s role in social behaviors by showing that the excessive neuronal activation is restricted to the infralimbic sub-region. Two other brain areas implicated in social behavior, the piriform cortex and medial amygdala, show normal c-Fos activation, suggesting specificity of the aberrant activation pattern in the infralimbic mPFC. Further, the prelimbic sub-region of the mPFC shows abnormally low c-Fos activation in response to a novel object/non-social stimulus in APC cKOs, relative to control littermates. Intriguingly, APC cKOs show deficits in the novel object recognition cognitive task (Mohn et al., 2014). Our findings provide strong support for the importance of the infralimbic and prelimbic mPFC in discriminating between and gating the response to social vs. non-social stimuli, respectively.

Accumulating evidence suggests that E/I imbalance is a common pathophysiological change in autism (Lee et al., 2017), convergent across multiple risk factors. Optogenetic manipulations of E/I balance in the mPFC provide strong support for excessive excitation playing a critical role in autism-like social behavior (Yizhar et al., 2011; Selimbeyoglu et al., 2017). However, these studies did not discriminate between the prelimbic and infralimbic sub-regions, which have mostly distinct projections throughout the brain (Vertes, 2004). Here, we show increased excitation in the infralimbic pyramidal neurons in two different ways: excessive c-Fos activation in response to a social stimulus and increased frequency of mEPSCs. These changes are likely caused by the excessive β-catenin levels in the APC cKO pyramidal neurons, as high β-catenin is known to cause increases in both spine density and mEPSC frequency in cultured hippocampal neurons (Murase et al., 2002; Bamji et al., 2003; Yu and Malenka, 2004; Tai et al., 2007; Vitureira et al., 2012). Reports have identified cortical pyramidal neurons as an autism-relevant cell type based on convergent expression of ASD genes in both the human and mouse brain (Parikshak et al., 2013; Willsey et al., 2013), and our APC cKO mouse, using CamKIIα-Cre, predominantly targets forebrain glutamatergic neurons.

As further support that E/I imbalance is a common pathology in ASD, we show reduced expression levels of both PV and SST, proteins that characterize distinct inhibitory interneuron subtypes, in the APC cKO mPFC. Fast-spiking PV interneurons play a major role in regulating the activation and output of glutamatergic projection neurons in the cortex (Kawaguchi and Kubota, 1997; Beierlein et al., 2000; Kubota, 2014; Jiang et al., 2015). Reduced levels of PV have been seen in the prefrontal cortex of individuals with ASD and in mouse models expressing various ASD linked gene mutations (Gogolla et al., 2009; Peñagarikano et al., 2011; Saunders et al., 2013; Wöhr et al., 2015; Filice et al., 2016; Hashemi et al., 2017).

Altered SST levels are a less common feature in ASD. SST interneurons are known to inhibit PV interneurons and to impact the E/I balance in local circuits (Pfeffer et al., 2013; Xu et al., 2013; Urban-Ciecko and Barth, 2016). Similar to our finding of reduced SST in the APC cKO mPFC, two other studies suggest that SST interneuron dysfunction may link to autism-like behaviors. Mice with phosphatase PTEN cKO in cortical interneuron progenitors exhibit preferential loss of SST interneurons and deficits in social behavior (Vogt et al., 2015). Intriguingly, MeCP2 deletion restricted to either PV or SST interneurons leads to distinct neurological phenotypes: mice lacking MeCP2 in PV interneurons show motor, sensory, memory and social deficits, whereas mice lacking MeCP2 in SST interneurons exhibit stereotypies and seizures (Ito-Ishida et al., 2015).

Synergistic impairment of multiple neuron types: excitatory pyramidal neurons, PV interneurons and SST interneurons, are likely responsible for the neurological phenotypes of social deficits, repetitive behaviors, cognitive impairments and chronic seizures seen in APC cKO mice (Mohn et al., 2014; Pirone et al., 2017). Interneurons regulate the firing of pyramidal cells in ensembles, and the different interneuron subtypes may modulate ensemble activity for optimal task performance in different mPFC behaviors (Ferguson and Gao, 2018). Although our results suggest possible reductions in interneuronal input onto excitatory neurons in the infralimbic mPFC of APC cKOs, direct measurements of inhibitory currents are needed. Our future studies will further define the changes in interneuron diversity, function and integration into circuits in the mPFC of APC cKOs. Another interesting question to be addressed is the underlying mechanisms whereby malfunction of the β-catenin/Wnt pathway causes the cellular and circuit level changes.

Given the increasingly prominent role of the β-catenin/Wnt signaling network in ASD caused by numerous gene mutations, our findings provide important insights into relevant brain regions and cell types. We show increased excitation in the infralimbic sub-region of the mPFC and decreased PV and SST in APC cKO mice. E/I imbalance in the mPFC and reduced PV levels are emerging as shared changes across ASD linked to distinct risk factors (Gogolla et al., 2009; Peñagarikano et al., 2011; Yizhar et al., 2011; Saunders et al., 2013; Wöhr et al., 2015; Filice et al., 2016; Hashemi et al., 2017; Selimbeyoglu et al., 2017). Elucidating the common pathophysiological alterations in the brain is essential for defining targets with potential for effective therapeutic intervention to ameliorate autistic disabilities.

## Author Contributions

MJ, LR, TB, KM, AP and JA conceptualized the project and experiments. JA and LE performed the behavioral assays in coordination with DC, RW and MP who performed perfusions and immunostainings for the c-Fos experiments. AP and MP imaged the sections and NS quantified the changes, blinded to genotypes. AP also performed the PV and SST immunostaining, imaging and quantification. JK performed the electrophysiology experiments. JK and CD analyzed the data. JA and MJ wrote the manuscript and incorporated revisions from all authors.

## Conflict of Interest Statement

The authors declare that the research was conducted in the absence of any commercial or financial relationships that could be construed as a potential conflict of interest.
